# Histamine, leukotriene C4 and interleukin-2 increase antibody uptake into a human carcinoma xenograft model.

**DOI:** 10.1038/bjc.1991.416

**Published:** 1991-11

**Authors:** T. W. Hennigan, R. H. Begent, T. G. Allen-Mersh

**Affiliations:** Department of Surgery, Charing Cross and Westminister Medical School, London, UK.

## Abstract

**Images:**


*Br. J. Cancer* (1991), **64**, 872–874 © Macmillan Press Ltd., 1991

### Histamine, leukotriene C4 and interleukin-2 increase antibody uptake into a human carcinoma xenograft model

T.W. Hennigan[1], R.H. J. Begent[2] & T.G. Allen-Mersh[1]

[1]*Department of Surgery, Charing Cross and Westminster Medical School, Fulham Palace Road, London W6 8RF;* [2]*Cancer Research Campaign Clinical Research Laboratories, Department of Clinical Oncology, Royal Free Hospital School of Medicine, Rowland Hill Street, London NW3 2PF, UK.*

**Summary** Systemically administered radiolabelled anti-tumour antibody is ineffective in treating the majority of patients with liver metastasis from colorectal carcinoma. We have assessed whether agents which increase capillary permeability can increase tumour uptake of antibody isotope conjugate. We developed a xenograft model of colorectal carcinoma using an antibody directed against carcinoembryonic antigen (CEA). Tumours were grown subcutaneously in the hind limbs of athymic rats to derive their circulation from the femoral artery. Cannulae were placed in the common iliac artery and iliolumbar vein. Antibody was delivered systemically, regionally and regionally with histamine, leukotriene $C_4$ and interleukin-2.

Regionally administered anti-CEA antibody resulted in a significantly greater ($P = 0.004$) tumour to normal tissue ratio (1.66, s.d. = 0.68) compared to systemically administered antibody (1.25, s.d. = 0.73). The addition of vasoactive drugs produced an approximately 3-fold increase with an increase to a mean tumour:liver ratio of 3.24 (s.d. = 1.39) for histamine ($P < 0.001$ compared to systemic delivery), 3.21 (s.d. = 1.13, $P < 0.001$) for leukotriene $C_4$ and 3.80 (s.d. = 1.53, $P < 0.001$) for interleukin-2.

The addition of histamine significantly ($P = 0.004$) increased the mean tumour to liver ratio (1.73, s.d. = 0.44) of non-specific antibody uptake compared with either systemic (1.12, s.d. = 0.24) or regional delivery (1.25, s.d. = 0.54) of non-specific antibody alone.

Increasing tumour capillary permeability can produce a significant clinically useful increase in tumour uptake of antibody-isotope conjugate.

Treatment of disseminated colorectal carcinoma using systemically administered anti-tumour antibody labelled with a therapeutic dose of iodine 131 has produced poor responses (Begent *et al.*, 1989) because of low tumour antibody uptake. The uptake of antibody from the circulation depends on intravascular dose, tumour blood flow (Sands *et al.*, 1986) and capillary permeability (Sands *et al.*, 1988). In the case of antibody macromolecules (IgG, molecular weight 150,000 daltons) capillary permeability is an important limitation to tissue uptake (Halpern *et al.*, 1986; Sands *et al.*, 1985).

We assessed the value of increasing capillary permeability by using agents known to increase capillary permeability in normal tissues. The agents selected were the vasodilator autocoid histamine (Douglas, 1980), the vasoconstrictor eicosanoid leukotriene $C_4$ (Frolich & Yoshizawa, 1987; Goetz *et al.*, 1987; Badr, 1984) and the biological response modifier interleukin-2 (Rosenstein *et al.*, 1986) which has minimal influence on vasomotor tone. Since these agents all have a short half life, they were administered regionally to enhance local effect and reduce systemic toxicity.

To assess whether these agents could increase uptake of antibody, we first developed a xenograft model which allows cannulation of tumour circulation and regional infusion of antibody to a human colorectal carcinoma expressing carcinoembryonic antigen (CEA).

### Methods

Two antibody conjugates were prepared: specific anti-CEA antibody (A5B7) labelled with iodine 131 and non-specific antibody (anti-HCG antibody, SB10) labelled with iodine 125. Both were labelled using the chloramine T method (Greenwood *et al.*, 1963).

Genetically immunocompromised rats (150–200 g) were maintained in a negative pressure isolator (Isotec, Bicester) and fed on sterile water and irradiated feed ad libitum. One million cells from a human colon cancer cell line (LS174T) in 0.1 ml of complete medium (Gibco, Uxbridge) were injected subcutaneously into the mid-thigh of the hind limb. Once solid tumour had grown the tumour line was serially passaged by implanting diced fragments of these tumours (mean weight 15 mg) into the subcutaneous fat of the hind limb. Arteriography confirmed that the tumours grew to derive their blood supply from the femoral artery (Figure 1).

**Figure 1** Arteriogram performed through the femoral artery cannula. The tumour (T) has filled with contrast supplied directly via the femoral artery cannula (arrow). The bladder has been filled with contrast excreted via the kidneys.

Correspondence: T.W. Hennigan.
Received 25 February 1991; and in revised form 20 June 1991.

When 300–600 mg tumours, determined by calliper measurements and volume approximation (Staab & Anderer, 1982) had grown the animal was anaesthetised the two cannulae (Portex 0.4 mm internal diameter, Arterial Medical Supplies, London) were inserted via a laparotomy incision. The first ('venous' or 'systemic') cannula was inserted into the right iliolumbar vein so that its tip lay at the junction of the iliolumbar vein and the inferior vena cava to gain access to the systemic circulation. The second ('arterial' or 'regional') cannula was passed retrogradely into the right common iliac artery so that its tip lay at the aortic bifurcation to gain access to the arterial supply to the contralateral tumour-bearing limb.

Antibody was then administered. In 24 animals the infusion of anti-carcinoembryonic antigen antibody (A5B7) labelled with iodine 131 was delivered into the systemic cannula with an infusion of saline delivered regionally. In 24 animals the situation was reversed and the conjugate delivered regionally with a control infusion of saline given systemically. In 30 animals the conjugate was mixed with histamine (Sigma, 5 mg $kg^{-1}$, $n = 12$), leukotriene $C_4$ (Sigma, 25 mcg/animal, $n = 8$) or interleukin-2 (100,000 units/animal, $n = 10$) and then delivered regionally. The agents were prepared in degassed phosphate buffer at pH 7.4.

After a 1 h infusion, the abdomen was re-opened and the cannulae removed, carefully preserving flow to the tumour bearing limb and the animal was then recovered. After 48 h the animal was again anaesthetised and the liver and tumour excised and weighed. Liver and tumour radioactivity was measured in a well counter to determine a tumour to liver gamma counts ratio.

### Results

The tumour to liver ratio of counts obtained in each group is shown in Figure 2.

### *Tumour to liver ratio of non-specific antibody*

Systemic administration of antibody via the inferior vena cava achieved a mean tumour to liver ratio of 1.12 (s.d. = 0.24). Regional delivery of antibody resulted in a tumour to liver ratio of 1.25 (s.d. = 0.54) which was not significantly greater (unpaired *t*-test) than after systemic delivery. The addition of histamine to the regionally delivered antibody produced a mean tumour to liver ratio of 1.73 (s.d. = 0.44) which was significantly ($P = 0.004$) greater than after systemic delivery.

### *Tumour to liver ratio of specific antibody*

Systemic administration of antibody via the inferior vena cava achieved a mean tumour to liver ratio of 1.25 (s.d. = 0.73). Regional delivery of antibody resulted in a mean tumour to liver ratio of 1.66 (s.d. = 0.68) which was significantly ($P = 0.048$) greater (unpaired *t*-test) than after systemic delivery. The addition of histamine to the regionally delivered antibody produced a mean tumour to liver ratio of 3.24 (s.d. = 1.39) which was significantly ($P < 0.001$) greater than after systemic delivery. Similarly, the mean tumour to liver ratio was 3.21 (s.d. = 1.13, $P < 0.001$) after administration of leukotriene $C_4$ and 3.80 (s.d. = 1.53, $P < 0.001$) for interleukin-2.

### Discussion

Regional delivery of labelled antibody into the arterial supply to the tumour-bearing limb produced only a small (33%) increase compared to systemic administration. Regional delivery is of greatest advantage where the agent administered has a high first pass extraction from the tumour vascular bed. Since the first pass extraction of antibodies is small (Halpern *et al.*, 1986), regional delivery would not be expected to provide a great increase in antibody uptake into tumour.

**Figure 2** There was a significant increase in tumour:liver counts if a capillary permeability agent was administered with specific or non-specific labelled antibody compared with labelled antibody alone.

Regional delivery was used primarily as a means of delivering short-acting vasoactive agents to the tumour circulation. We found that agents which increase capillary permeability in normal capillaries increased the uptake of antibody. Our study does not prove that the mechanisms of this increase was change in capillary permeability. However, each of the agents used has a different effect on microcirculation resistance and the effect observed was of a similar order of magnitude for all three agents. As increase in capillary permeability was the common factor, this is a likely explanation for the observed increase in antibody uptake.

This suggests that these agents are capable not only of influencing capillary permeability in normal tissues but also in the tumour circulation where capillaries are more rudimentary and varied in structure (Tannock & Steel, 1969; Vaupel, 1975). The finding that a similar effect was observed for non-specific antibody is compatible with enhancement of capillary permeability. However the smaller increase compared with specific antibody suggests that antigen-antibody binding contributed to the tumour labelled-antibody concentration.

The use of agents which increase capillary permeability in normal tissues can achieve an approximate 3-fold increase in specific antibody uptake. If radioimmunotherapy is to be effective, a 10-fold increase in antibody uptake relative to normal tissue may be required (Dykes *et al.*, 1987) although more modest increases may be of value in some patients (Begent *et al.*, 1989). The increase seen in this study, using regional delivery of agents which increase capillary permeability in normal tissues, is of sufficient magnitude to offer a prospect of therapeutic benefit if these findings were to be reproduced in man.

We would like to thank Pamela Pate for assistance with the care of animals, Dr R.B. Pedley for the provision of the LS174T cells and Mr R. Carpenter for the provision of the interleukin-2 and his guidance with the early development of the model.

Professor Begent and Dr R.B. Pedley were supported by the Cancer Research Campaign.

### References

BADR, K.F., BAYLIS, C., PFEFFER, J.M. & 6 others (1984). Renal and systemic haemodynamic responses to intravenous infusion of leukotriene $C_4$ in the rat. *Circ. Res.*, **54,** 492.

BEGENT, R.H.J., LEDERMAN, J.A., GREEN, A.J. & 7 others (1989). Antibody distribution and dosimetry in patients receiving radiolabelled antibody therapy for colorectal cancer. *Br. J. Cancer*, **60,** 406.

DOUGLAS, W.W. (1980). Histamine and 5-hydroxytryptamine (serotonin) and their antagonists. *The Pharmacological Basis of Therapeutics*. Gilman, A.G., Goodman, L.S. & Gilman, A. (eds), 6th edition, Macmillan: New York.

DYKES, P.W., BRADWELL, A.R., CHAPMAN, C.E. & VAUGHAN, A.T.M. (1987). Radioimmunotherapy of cancer: clinical studies and limiting factors. *Cancer Treat. Rev.*, **14,** 87.

FROLICH, J.C. & YOSHIZAWA, M. (1987). Renal vascular effects of leukotriene C4 in the isolated perfused kidney of the rat. *Br. J. Pharmacol.*, **92,** 311.

GREENWOOD, F.C., HUNTER, W.M. & GLOVER, J.S. (1963). The preparation of $^{131}$I-labelled human growth hormone of high specific radioactivity. *Biochem. J.*, **89,** 114.

GOETZ, A., DEININGER, F.D., CONZEN, P. & BRENDEL, W. (1987). Effects of FPL 55712 on leukotriene $C_4$/leukotriene $D_4$-induced vasoconstriction and macromelecular permeability. *Advances in Prostaglandin, Thromboxane and Leukotriene Research*. Vol. 17, Samuelsson, B., Paoletti, R. & Ramwell, P.W. (eds), Raven Press: New York.

HAPLERN, S.E., BUCHEGGER, F., SCHREYER, M. & MACH, J.-P. (1986). Effect of size of radiolabelled antibody and fragments on tumour uptake and distribution in nephrectomised mice. *J. Nucl. Med.*, **25,** 112.

ROSENSTEIN, M., ETTINGHAUSEN, S.E. & ROSENBERG, S.A. (1986). Extravasation of intravascular fluid mediated by the systemic administration of recombinant interleukin-2. *J. Immunol.*, **137,** 1735.

SANDS, H., JONES, P.L., NEACY, W.P., SHAH, S.A. & GALLAGHER, B.M. (1986). Site-related differences in the localisation of the monoclonal antibody OX7 in the SL2 and SL1 lymphomas. *Cancer Immunol. Immunotherap.*, **22,** 169.

SANDS, H., JONES, P.L., SHAH, S.A., PALME, D., VESSELLA, R.L. & GALLAGHER, B.M. (1988). Correlation of vascular permeability and blood flow with monoclonal antibody uptake by human Clouser and renal cell xenografts. *Cancer Res.*, **48,** 188.

SANDS, H., SHAH, S.A. & GALLAGHER, B.M. (1985). Vascular volume and permeability of human and murine tumours grown in athymic mice. *Cancer Lett.*, **27,** 15.

STAAB, H.J. & ANDERER, F. (1982). Growth of human colonic adenocarcinomas and development of serum CEA in athymic mice. I. Strict correlation of tumour size and mass with serum CEA concentration during logarithmic growth. *Br. J. Cancer*, **46,** 841.

TANNOCK, I.F. & STEEL, G.G. (1969). Quantitative techniques for the study of the anatomy and function of small blood vessels in tumours. *J. Natl Cancer Inst.*, **42,** 771.

VAUPEL, P. (1975). Inter-relationship between mean arterial pressure, blood flow and vascular resistance in solid tumour tissue of DS-carcinomesarcoma. *Experimentia*, **31,** 587.


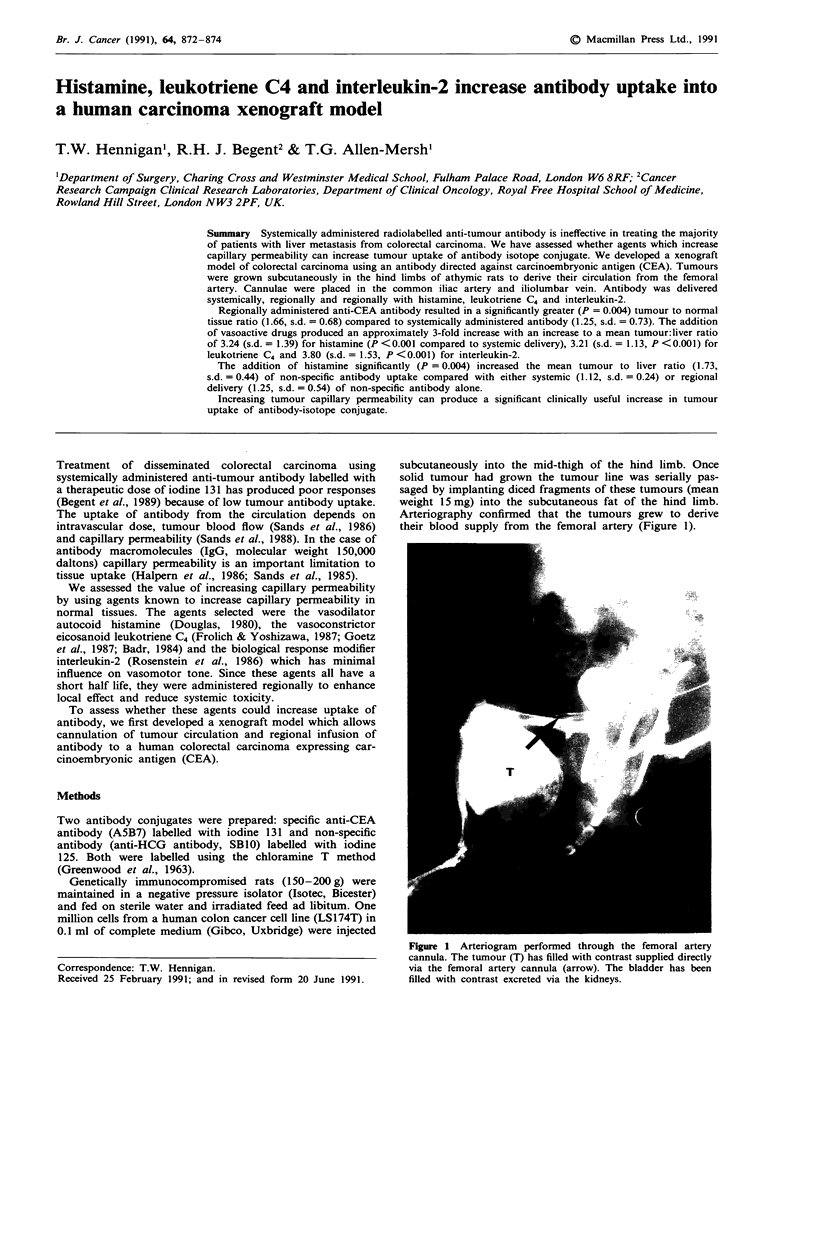

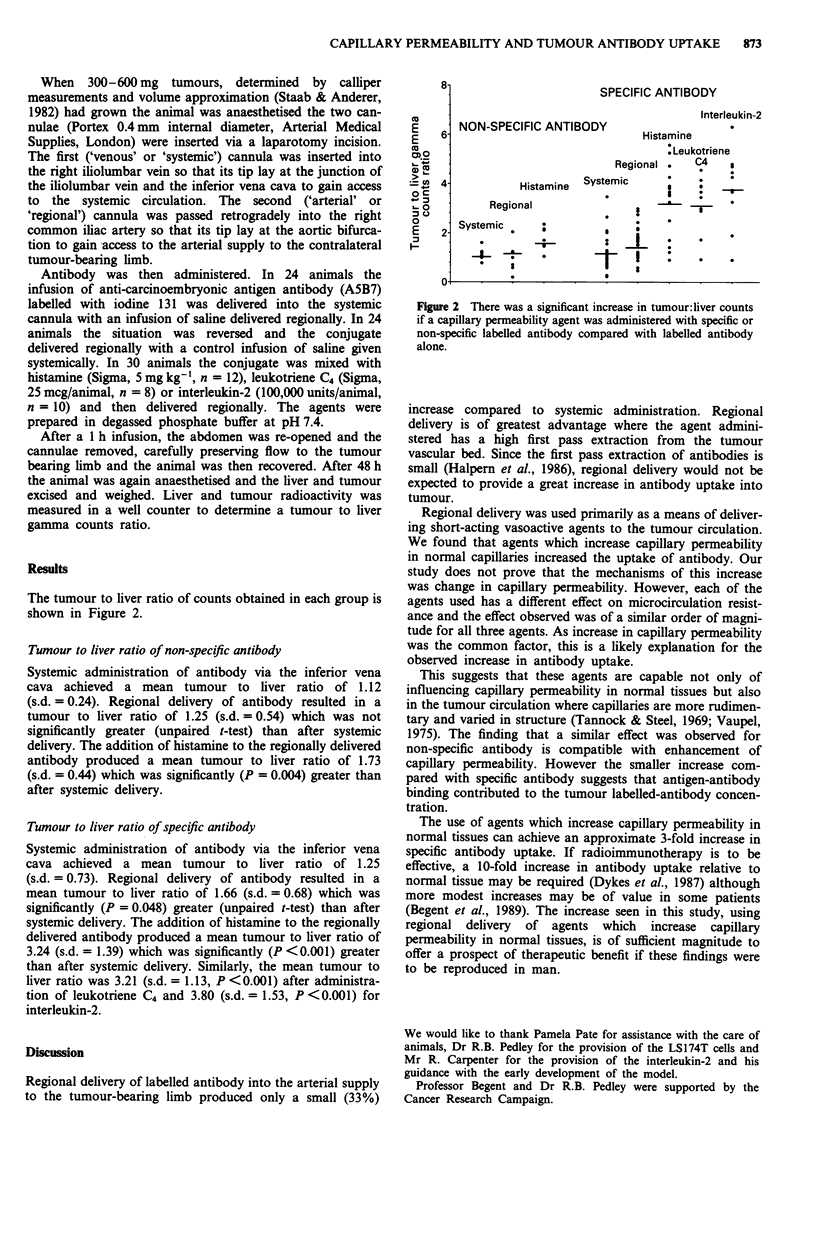

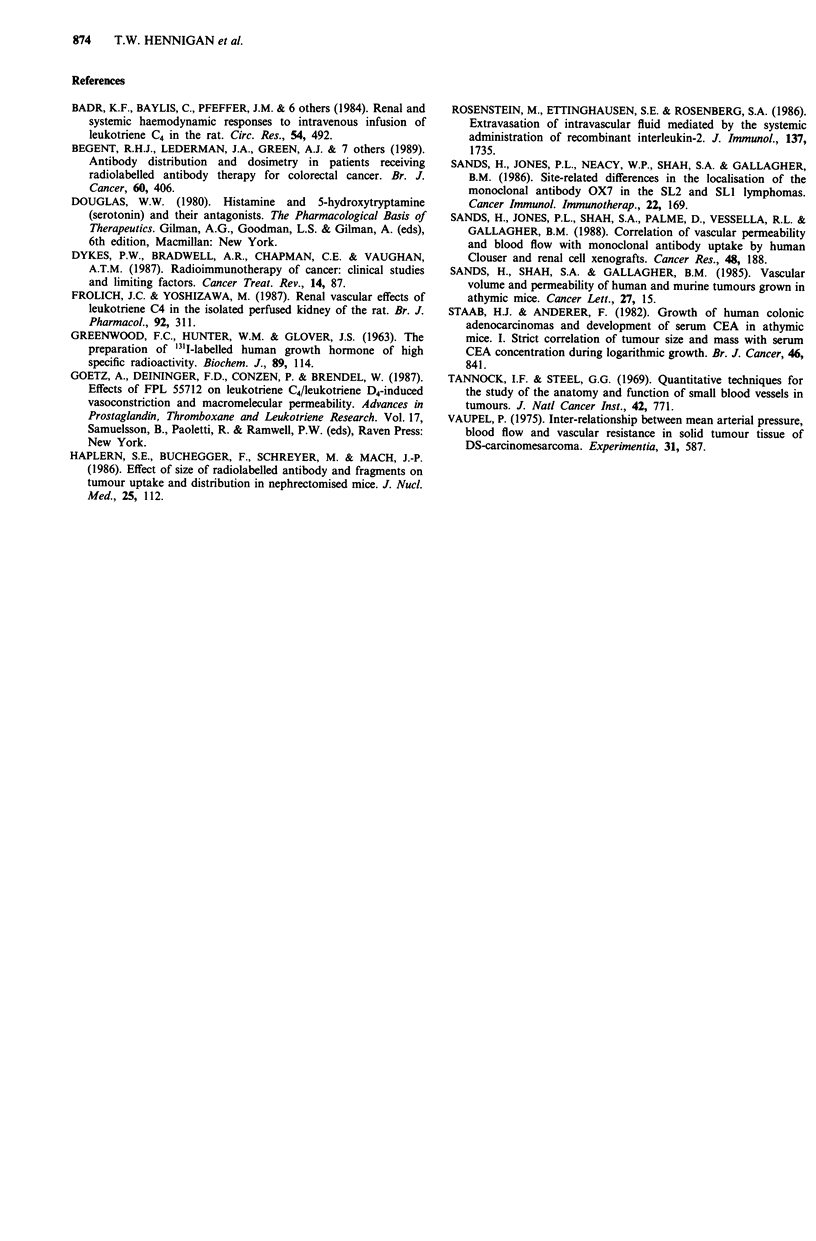


## References

[OCR_00283] Badr K. F., Baylis C., Pfeffer J. M., Pfeffer M. A., Soberman R. J., Lewis R. A., Austen K. F., Corey E. J., Brenner B. M. (1984). Renal and systemic hemodynamic responses to intravenous infusion of leukotriene C4 in the rat.. Circ Res.

[OCR_00286] Begent R. H., Ledermann J. A., Green A. J., Bagshawe K. D., Riggs S. J., Searle F., Keep P. A., Adam T., Dale R. G., Glaser M. G. (1989). Antibody distribution and dosimetry in patients receiving radiolabelled antibody therapy for colorectal cancer.. Br J Cancer.

[OCR_00298] Dykes P. W., Bradwell A. R., Chapman C. E., Vaughan A. T. (1987). Radioimmunotherapy of cancer: clinical studies and limiting factors.. Cancer Treat Rev.

[OCR_00303] Frölich J. C., Yoshizawa M. (1987). Renal vascular effects of leukotriene C4 in the isolated perfused kidney of the rat.. Br J Pharmacol.

[OCR_00308] GREENWOOD F. C., HUNTER W. M., GLOVER J. S. (1963). THE PREPARATION OF I-131-LABELLED HUMAN GROWTH HORMONE OF HIGH SPECIFIC RADIOACTIVITY.. Biochem J.

[OCR_00327] Rosenstein M., Ettinghausen S. E., Rosenberg S. A. (1986). Extravasation of intravascular fluid mediated by the systemic administration of recombinant interleukin 2.. J Immunol.

[OCR_00333] Sands H., Jones P. L., Neacy W. P., Shah S. A., Gallagher B. M. (1986). Site-related differences in the localization of the monoclonal antibody OX7 in SL2 and SL1 lymphomas.. Cancer Immunol Immunother.

[OCR_00339] Sands H., Jones P. L., Shah S. A., Palme D., Vessella R. L., Gallagher B. M. (1988). Correlation of vascular permeability and blood flow with monoclonal antibody uptake by human Clouser and renal cell xenografts.. Cancer Res.

[OCR_00345] Sands H., Shah S. A., Gallagher B. M. (1985). Vascular volume and permeability of human and murine tumors grown in athymic mice.. Cancer Lett.

[OCR_00350] Staab H. J., Anderer F. A. (1982). Growth of human colonic adenocarcinoma and development of serum CEA in in athymic mice. I: Strict correlation of tumour size and mass with serum CEA concentration during logarithmic growth.. Br J Cancer.

[OCR_00357] Tannock I. F., Steel G. G. (1969). Quantitative techniques for study of the anatomy and function of small blood vessels in tumors.. J Natl Cancer Inst.

[OCR_00362] Vaupel P. (1975). Interrelationship between mean arterial blood pressure, blood flow, and vascular resistance in solid tumor tissue of DS-carcinosarcoma.. Experientia.

